# Wakeful resting and listening to music contrast their effects on verbal long-term memory in dependence on word concreteness

**DOI:** 10.1186/s41235-022-00415-4

**Published:** 2022-09-03

**Authors:** Markus Martini, Jessica R. Wasmeier, Francesca Talamini, Stefan E. Huber, Pierre Sachse

**Affiliations:** 1 University of Innsbruck, Innrain 52, 6020 Innsbruck, Austria; 2 Department of Psychology, University of Innsbruck, 6020 Innsbruck, Austria

**Keywords:** Wakeful resting, Listening to music, Memory consolidation, Concreteness, Imageability, Interference

## Abstract

Wakeful resting and listening to music are powerful means to modulate memory. How these activities affect memory when directly compared has not been tested so far. In two experiments, participants encoded and immediately recalled two word lists followed by either 6 min wakefully resting or 6 min listening to music. The results of Experiment 1 show that both post-encoding conditions have a similar effect on memory after 1 day. In Experiment 2, we explored the possibility that less concrete words, i.e. lower in imageability than in Experiment 1, are differently affected by the two post-encoding conditions. The results of Experiment 2 show that, when words are less concrete, more words are retained after 1 day when encoding is followed by wakeful resting rather than listening to music. These findings indicate that the effects of wakeful resting and listening to music on memory consolidation are moderated by the concreteness of the encoded material.

**Supplementary Information:**

The online version contains supplementary material available at 10.1186/s41235-022-00415-4.

## Significance statement

The phase immediately after learning plays a fundamental role in the life of new memories. When new memories are formed, they continue to be processed by our brain even when the learning event is already over. Studies indicate that neurophysiological processes responsible for establishing enduring memories are vulnerable to disruption especially immediately after learning. Accordingly, studies show that a short break immediately after learning, with closed eyes in a relaxing state, can support memory, whereas task engagement, i.e. doing something cognitively demanding, can disrupt memory. Researchers are interested in identifying which post-learning activities support memory and which disrupt memory. Here, we report two experiments investigating which impact a short resting phase has on memory in younger adults compared to a short phase of listening to music after learning a word list. Our findings indicate that for more concrete learning material—words high in imageability like “cup” or “umbrella”—resting and listening to music yield similar proportions of remembered words after 1 day. However, when the learning material gets less concrete—when words are low in imageability like “truth” or “hunch”—more words are remembered after 1 day when participants rested compared to when participants listened to music. The reported study provides new insights into how two familiar activities affect verbal memory formation in dependence of the concreteness of the learning material.

## Introduction

Numerous behavioural and neuroscientific findings indicate that new memories encoded while awake continue to be processed ‘off-line’ immediately after their acquisition, and that during this temporal window they are modifiable, i.e. the post-encoding activity can influence how much is remembered later (Dewar et al., 2007; Dudai et al., 2015; McGaugh, 2018; Tambini & Davachi, 2019; Wamsley, 2019; Wixted, 2005, 2010). The study presented here investigated the effects of two familiar post-encoding ‘activities’: wakeful resting and listening to music. Both activities are powerful means to modulate memory formation (Dewar et al., 2007; Judde & Rickard, 2010). However, no study so far investigated these two post-encoding activities in direct comparison to contrast their effects on memory.

### Wakeful resting after encoding

Wakeful resting can be described as a state during which outwardly focussed attention is reduced, while cognitive resources are devoted to spontaneous, internally focussed mind wandering processes, including thinking, planning, dreaming, and imagination (Wamsley, 2019; see also Smallwood & Schooler, 2015). A common methodological approach to investigate the impact of wakeful resting on memory is to contrast its effects with a task in which participants are cognitively loaded. For example, participants encode and immediately recall a word list. The critical manipulation takes place immediately after the memory test. Participants either wakefully rest for several minutes in a relaxing state and closed eyes, or perform a cognitively demanding task for several minutes, like spotting differences in images or solving complex problems. At the end of the experiment, participants’ memory on the word list is tested again in a final, unannounced memory test. With this methodological approach, the majority of existing studies find that memory retention is higher when encoding is followed by a brief period of wakeful resting compared to when encoding is followed by performing a new task (Wamsley, 2019; cf Martini & Sachse, 2020). This basic finding has been replicated several times with different encoding materials (verbal: Brokaw et al., 2016; Dewar et al., 2012; visuospatial: Craig et al., 2015; procedural: Humiston & Wamsley, 2018), distractor tasks (Dewar et al., 2007), retention intervals (minutes: Mercer, 2015; days: Dewar et al., 2012), and age groups (children: Martini et al., 2019a; older adults: Dewar et al., 2012; Martini et al., 2019b). One of the primary explanations why wakeful resting benefits memory is that states of reduced interference support memory consolidation—a family of neurophysiological processes transforming new memory representations into lasting ones (Dudai et al., 2015; Hasselmo, 1999). Memory consolidation during the awake state seems thereby to be similar to memory consolidation during sleep. For instance, Brokaw et al. (2016) found that improved memory was associated with an increase in slow oscillatory brain activity, an EEG signature proposed to facilitate memory consolidation also during sleep. It is further assumed that during memory consolidation, the newly encoded information is repeatedly reactivated (Bergmann & Staresina, 2017; Dudai et al., 2015; Tambini & Davachi, 2019). A repeated reactivation helps a new memory representation to get stabilized and to become less prone to interference over time. Studies indicate that new memories are vulnerable to consolidation interference especially immediately after encoding (Robertson, 2012; Wixted, 2005). Consequently, during a period of wakeful rest, our brain finds optimal conditions to reactivate and consolidate new memories while task engagement can disrupt reactivation and consolidation processes potentially leading to a loss of these new memories (Dewar et al., 2007, 2012; Mednick et al., 2011; Tambini & Davachi, 2019; Wamsley, 2019). The magnitude of the reactivation seems thereby related to improved memory. Tambini et al. (2010) found enhanced functional connectivity between task-relevant brain areas (hippocampus and a portion of the lateral occipital complex) during rest following a task with high subsequent memory. Furthermore, they found that the magnitude of the correlation between the connectivity of the learning relevant brain areas during rest predicted individual differences in a later memory test (see also Buch et al., 2021). These findings are in good agreement with results from sleep research (Schapiro et al., 2018).

### Listening to music after encoding

Music can be a powerful means to modulate memory (Ferreri & Verga, 2016). Findings on the impact of listening to music on memory range from memory-enhancing effects (Rauscher et al., 1993; Thompson et al., 2001), to memory detrimental effects (Iwanaga & Ito, 2002; Thompson et al., 2011;), to no-effects (Jäncke & Sandmann, 2010; Nguyen & Grahn, 2017)—with various factors modulating the nature and magnitude of musical reactivity (e.g. Husain et al., 2002). The majority of these studies investigated the impact of music on memory when music was presented before new encoding or during new encoding. However, we are aware of only three studies so far investigating the impact of music on memory when music was presented immediately *after* new encoding (Greene et al., 2010; Judde & Rickard, 2010; Rickard et al., 2012, Experiment 2). Music is eminently suitable to induce and modulate both, arousal and emotion (Baumgartner et al., 2006; Greene et al., 2010; VanderArk & Ely, 1992; see also Mather & Sutherland, 2011; McGaugh, 2015, 2018). Therefore, it appears reasonable to speculate that music might have an intrinsic capacity to modulate memory consolidation. Greene et al. (2010) showed that the interaction of mood and arousal induced by music following encoding of abstract shapes enhanced recognition memory. Memory was enhanced both in a positive mood and high arousal state as well as in a negative mood and low arousal state, both relative to a positive mood and low arousal state or a negative mood and high arousal state. The study of Judde and Rickard (2010) showed that recognition of neutral words after 7 days was significantly enhanced, regardless of valence, when music was presented 20 min after encoding, yet not when it was presented immediately or 45 min after encoding, compared to a control condition in which participants just encoded and immediately recalled the word list. Findings of Rickard et al., (2012; Experiment 2) showed that more details of an emotional story were retained over 7 days when learning was followed by background sound or arousing music (marginally significant), and reduced when learning was followed by relaxing music (compared to a neutral story condition).

### The present study

The present study aimed at contrasting the effects of wakeful resting and listening to music on memory. Participants encoded and immediately recalled two word lists. Recall of one word list was followed by 6 min of wakeful resting; recall of the other word list was followed by 6 min of listening to music. Memories for both word lists were tested again after 24 h.

Findings from earlier studies indicate that listening to music immediately after encoding either has no impact on memory or memory-enhancing effects (Greene et al., 2010; Judde & Rickard, 2010; Rickard et al., 2012)—the latter effect is potentially modulated by a change in the (emotional) arousal state (see McGaugh, 2015, 2018) or the novelty of the stimulus (Moncada &Viola, 2007; but see also Wang & Morris, 2010). However, those few existing studies, which investigated the impact of listening to music on memory when presented immediately after the encoding of new information did not directly compare a wakeful resting condition to a listening to music condition.

The majority of findings from research on wakeful resting indicate that memory retention is supported by a brief period of post-encoding wakeful resting compared to a post-encoding period in which (i) a mentally effortful new task is performed, whereby the interfering material does not have to be similar to the to-be-remembered material (Dewar et al., 2007, 2014; Martini et al., 2019a; for conflicting results see Martini et al., 2020; Varma et al., 2017), or (ii) attention is inwardly focussed, for example, when rich autobiographical retrieval/future imagination is stimulated by short auditory sound cues (Craig et al., 2014; Varma et al., 2018). These findings resonate with the view that any condition that results in reduced interference, i.e. during which no new memories are formed (e.g. slow-wave sleep, benzodiazepines, acetylcholine antagonists) support memory consolidation (Mednick et al., 2011; see also Tambini & Davachi, 2019; Dudai et al., 2015; Wamsley, 2019; Hasselmo, 1999; Hasselmo & McGaughy, 2004). Based on the previous outlines, we therefore expected to find that more words are retained over 1 day period when encoding is followed by wakeful resting compared to when encoding is followed by listening to music.

## Experiment 1

### Method

#### Participants

We calculated the sample size required to observe a significant wakeful resting effect based on the pwr package (Champely et al., 2020) using an alpha of 0.05, power of 0.80, and a medium effect size. We found the minimum required sample size to be twenty-six participants. Forty university students took part in the experiment in exchange for course credit (31 female; mean age = 23.08 years; SD = 5.68; age range = 18–55 years). All participants gave informed consent to take part in the study.

#### Materials and procedure

The experiment included two testing sessions, denoted as Session 1 and Session 2 in the remainder of this work. The two sessions were separated by 1 day. We applied a repeated measures design with the within-subjects factors of post-encoding activity (wakeful resting vs. listening to music) and time of recall (immediate vs. after 1 day).

#### Session 1

Session 1 included two word-encoding phases, which occurred one after the other. Each word-encoding phase consisted of (a) a visual presentation of one of two word lists (with instructions to remember as many words as possible for a subsequent immediate recall), (b) immediate free recall of the word list, (c) a ~ 6 min post-encoding activity delay, which participants spent either wakefully resting or listening to music, and (d) answering of post-encoding activity questions.

The two word lists consisted of 15 German words each. Words were taken from the Leipzig Affective Norms for German (LANG; Kanske & Kotz, 2010). The LANG consists of one-thousand German nouns that have been rated on the dimension of emotional valence (9 points scale ranging from negative, neutral to positive), arousal (9 points scale ranging from high arousing to low arousing), and concreteness (9 points scale ranging from concrete to abstract). We selected only words from the LANG database falling within ± 1SD of the reported mean LANG rating scores for the dimensions valence (*M* = 5.07, SD = 1.20), arousal (*M* = 2.97, SD = 1.96), and concreteness (*M* = 2.07, SD = 1.38). Words were mono- and bisyllabic and consisted of four to six letters (e.g. “Tasse[cup]”, “Schirm[umbrella]”, “Kran[crane]”, “Regal[shelf]”, “Angel[rod]”). We created the word lists such that each word started with a different first letter. Words were presented one by one, in a pseudo-randomized order, for 2 s in the middle of the screen (black against a white background). In the recall phase, participants were asked to write down as many words from the previously presented word list as possible, in any order they wanted, on a blank sheet of paper, within 45 s.

During the wakeful resting delay, participants were asked to rest quietly, in a relaxed sitting position with their eyes closed in the darkened testing room for 5.45 min. The experimenter rested with the participants. Participants wore no headphones during the wakeful resting phase.

During the listening to music delay, participants listened to five music excerpts. A music excerpt comprised the chorus of a piece of music lasting about 60 s. Participants were asked to listen to and engage in the music with their eyes closed in the darkened testing room for 5.45 min. The selected music excerpts consisted of vocal songs, between 150 and 180 bpm fast (Smallpools: “American love”; Aloe Blacc: “Can you do this?”; We the Kings: “Kids in the moonlight”; KSHMR & Yves feat. Krewella: “No regrets”; Neon Trees: “Everybody talks”). Participants listened to the music via headphones. Music excerpts were presented in the same sequential order, without breaks, to all participants, at a comfortable volume level. The five pieces of music were selected by six student raters. In an online survey, they were asked to listen to twenty pieces of music (all ≥ 150 bpm), each presented for 30 s while their eyes should be closed. The raters answered questions on arousal, valence, familiarity, perceived subjective speed, and liking. Music from the genres pop, alternative, and dance was presented.

Emotional arousal was assessed before each word-list encoding phase and directly after each post-encoding activity (“At the moment I feel emotionally aroused”; 1 = “strongly disagree” to 6 = “strongly agree”; ”In the previous wakeful resting/listening to music condition I felt emotionally aroused”; 1 = “strongly disagree” to 6 = “strongly agree”). Additionally, following each post-encoding activity we asked participants whether they rehearsed the previously presented word list or parts of it (e.g. “In the previous resting phase I rehearsed the word list or parts of it”; 1 = “not at all” to 6 = “very often”), and whether they fell asleep (e.g. “In the previous resting phase I fell asleep”; yes/no). No explicit explanation of what defines a rehearsal activity was given to participants. Additionally, we asked participants about their thoughts and feelings during the delay condition, measured with the Amsterdam Resting State Questionnaire (ARSQ 2.0; Diaz et al., 2014) in addition to seven self-generated questions. The ARSQ measures mind wandering activity with 30 items via a five-point Likert scale (1 = “completely disagree” to 5 = “completely agree”) resulting in ten factors (discontinuity of mind, theory of mind, self, planning, sleepiness, comfort, somatic awareness, health concern, visual and verbal thought; for details see Diaz et al., 2014). With our self-generated questions, we were interested in whether participants’ thoughts were related to the past, present, or future (“I thought about something in the past/present/future; 1 = “strongly disagree” to 5 “strongly agree”) and their emotional connotation (“I thought about something that brings sorrow/happiness/anger/sadness; 1 = “strongly disagree” to 5 “strongly agree”).

The order of the two post-encoding activity conditions was counterbalanced across participants. Twenty participants were in the ‘first wakeful resting-then listening to music’ condition and twenty participants in the ‘first listening to music-then wakeful resting’ condition. Each participant had a partition on her/his right and left and the distance between participants was about two meters to increase privacy and decrease distraction. Participants were tested in groups of one to maximum four participants. Between the two word-encoding phases, participants were instructed to fill out questionnaires regarding participants’ personality, sensitivity, and music preference for about 10 min. Data from these questionnaires are not reported in the present study.

#### Session 2

Session 2 started between 19 and 31 h after Session 1 (*M* = 25 h). In Session 2, participants were asked to recall both word lists, beginning with the word list that had been presented first in Session 1. Participants were asked to write down as many words as possible, within 90 s per word list, in any order they wanted on a blank sheet of paper.

### Analysis of the data

#### Memory performance

Our measure of participants´ memory performance for each word list recall was the number of words correctly recalled (15 per word list). Delayed (1-day) recall test performance was scored such that a recalled word was rated correctly regardless of whether the participant correctly assigned the recalled word to the correct word list. For instance, if a participant remembered the word ‘corn’ from list 1 but mismatched the word to list 2 it was scored correctly recalled. To examine how much of the immediately recalled words were retained over 1 day in each post-encoding activity condition (wakeful resting vs. listening to music), we computed for each participant a percentage-retention score for each condition. Retention scores were calculated by dividing the number of words recalled correctly after 1 day by the number of words recalled correctly at immediate recall in Session 1 and multiplying the quotient by 100 (e.g. Dewar et al., 2012). Memory retention scores were statistically analysed using a paired-samples t test.

#### Subjective rehearsal ratings

Participants’ responses to the question of how often they rehearsed the word list or parts of it during wakeful resting and listening to music were compared using a Wilcoxon signed-rank test. Relations between participants’ rehearsal ratings and memory retention scores in the respective post-encoding activity condition (wakeful resting vs. listening to music) were analysed using Spearman correlations.

#### Subjective emotional arousal ratings

To measure the change in emotional arousal in the respective post-encoding activity condition (wakeful resting vs. listening to music) we calculated an emotional arousal change score for each participant. Emotional arousal change scores were calculated by dividing participant’s emotional arousal rating before encoding a word list divided by the emotional arousal rating after the respective post-encoding activity (wakeful resting vs. listening to music). Differences in the emotional arousal change scores between the post-encoding activity conditions were analysed using a Wilcoxon signed-rank test. Correlational analyses with memory retention scores in the respective post-encoding activity condition (wakeful resting vs. listening to music) were analysed using Spearman correlations.

#### Thought activity

For the ARSQ questionnaire, we followed the scoring method described in Diaz et al. (2014). Data from the self-generated items were analysed based on participants’ raw scores. Descriptive statistics as well as inference statistical analyses using Spearman correlations between the ARSQ dimensions and our mind wandering items with memory retention over 1 day can be found in the Supplements (Additional file 1: Table 1).

Statistical analyses were conducted using JASP (JASP Team, 2020). The alpha level was set at < 0.05.

### Results

#### Memory performance

**Fig. 1:**
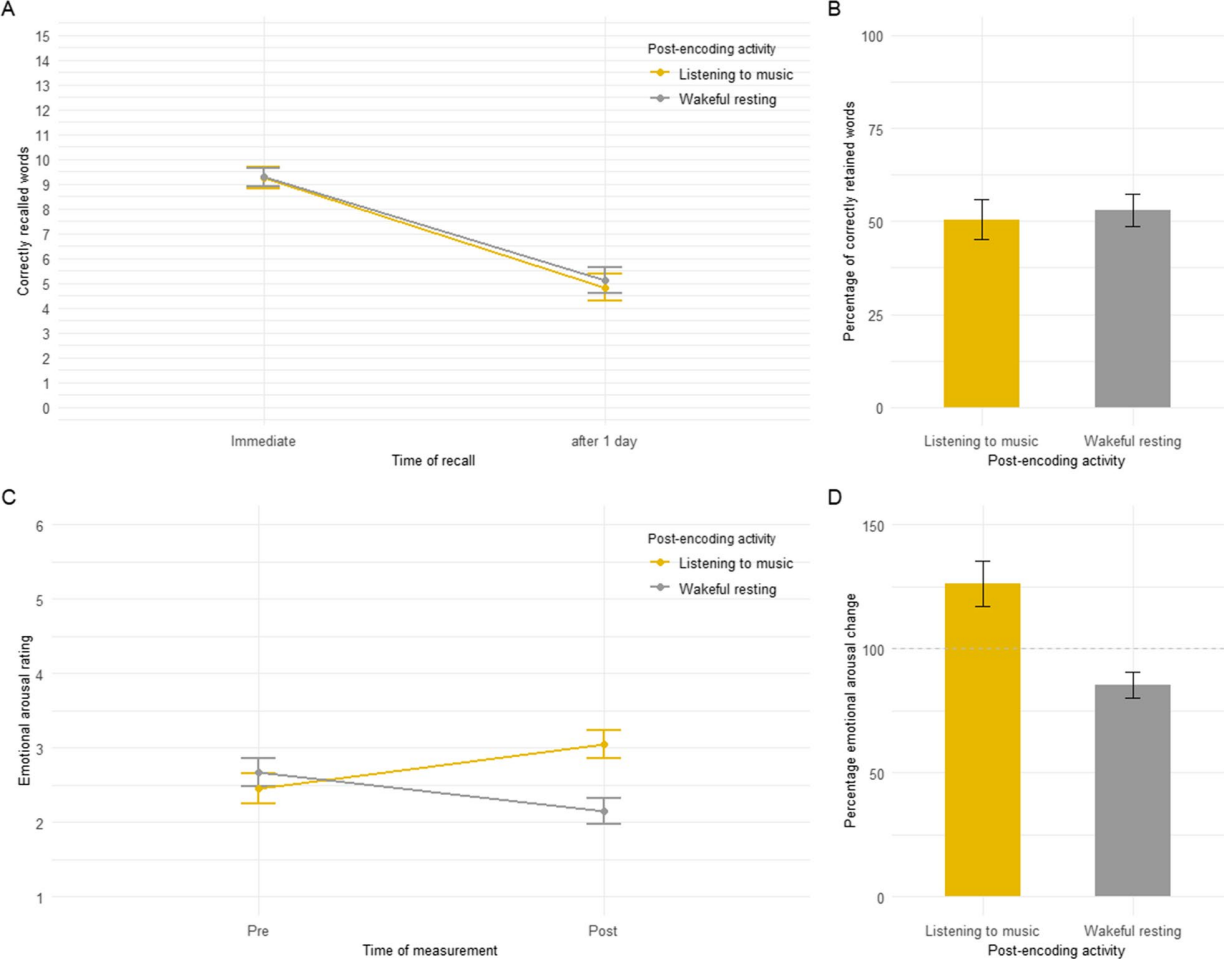
**A** Mean number of words correctly recalled (out of 15 per word list) as a function of the post-encoding activity (wakeful resting [grey] vs. listening to music [yellow]) and time of recall (immediate vs. after 1 day). **B** Mean retention scores in percent as a function of post-encoding activity (wakeful resting [grey] vs. listening to music [yellow]). Retention scores were calculated by dividing the number of correctly recalled words in Session 2 by the number of correctly recalled words in Session 1 and multiplying the quotient by 100. **C** Mean emotional arousal ratings as a function of the post-encoding activity (wakeful resting [grey] vs. listening to music [yellow]) and time of measurement (before learning the word list [pre] vs. after the post-encoding activity [post]). **D** Percentage emotional arousal change scores as a function of post-encoding activity (wakeful resting [grey] vs. listening to music [yellow]). Percentage emotional arousal change scores were calculated by dividing participant’s emotional arousal rating before encoding a word list divided by the emotional arousal rating after the respective post-encoding activity (wakeful resting vs. listening to music) multiplied by 100. Dashed helpline: 100% indicates no change in the mean pre- to post emotional arousal ratings. Error bars represent standard errors of the mean

Mean memory performance at the two recall times in the two post-encoding activity conditions (wakeful resting vs. listening to music) is presented in Fig. 1A, which depicts the raw number of words recalled, and in Fig. 1B, which depicts the percentage-retention scores. As shown in Fig. 1B, participants retained a similar amount of words after 1 day in the wakeful resting condition and listening to music condition, *t*(39) = 0.74, *p* = 0.463, *d* = 0.117. The order of the post-learning condition (first wakeful resting [*M* = 0.54, SD = 0.28]-then listening to music [*M* = 0.44, SD = 0.30] versus first listening to music [*M* = 0.56, SD = 0.30]-then wakeful resting [*M* = 0.52, SD = 0.27]) revealed no significant effect for post-encoding activity, *F*(1,38) = 0.58, *p* = 0.449, *η*_*p*_^*2*^ = 0.015, order, *F*(1,38) = 0.41, *p* = 0.53, *η*_*p*_^*2*^ = 0.011, and the post-encoding activity*order interaction, *F*(1,38) = 3.42, *p* = 0.072, *η*_*p*_^*2*^ = 0.083. The same pattern of results was obtained when the raw number of words recalled was analysed (see Fig. 1A and Supplements, Additional file 2: Experiment 1 - Analyses of the raw number of words recalled).

#### Subjective rehearsal ratings

Participants’ rehearsal ratings indicate that words were only rarely rehearsed during the respective post-encoding activity condition (wakeful resting: *M* = 2.33, SD = 1.46; listening to music: *M* = 1.90, SD = 1.41). Rehearsal ratings did not differ significantly between the wakeful resting condition and the listening to music condition, *W* = 265.00, *p* = 0.153, *r*_rank-biserial_ = 0.305. Rehearsal ratings in the wakeful resting condition were not significantly correlated with memory retention in the wakeful resting condition, *r*_Spearman_ = 0.245, *p* = 0.128 (see Additional file 3: Fig. 3, Supplements). Rehearsal ratings in the listening to music condition were significantly correlated with memory retention in the listening to music condition, *r*_Spearman_ = 0.46, *p* = 0.003 (see Additional file 3: Fig. 3, Supplements). Exclusion of those participants responding to the rehearsal question in the listening to music condition in a range between 5 and 6 (*n* = 3) and reanalysing the differences in memory retention scores between the wakeful resting condition and listening to music condition did not change our previously reported pattern of results in the ‘Memory performance’ section.

#### Subjective arousal ratings

Mean arousal ratings at the two recall times in the two post-encoding activity conditions (wakeful resting vs. listening to music) are presented in Fig. 1C, which depicts the raw emotional arousal ratings, and in Fig. 1D, which depicts the emotional arousal change scores (for a description see Methods section). As shown in Fig. 1D, emotional arousal change increased in the listening to music condition (> 100%) and decreased in the wakeful resting condition (< 100%), *W* = 50.50, *p* < 0.001, *r*_rank-biserial_ = − 0.809. Emotional arousal change in the wakeful resting condition was not significantly correlated with memory retention in the wakeful resting condition, *r*_Spearman_ = 0.02, *p* = 0.897, and emotional arousal change in the listening to music condition was not significantly correlated with memory retention in the listening to music condition, *r*_Spearman_ = − 0.10, *p* = 0.545.

Participants’ thought activity was diverse within and between the respective post-encoding activity condition (wakeful resting vs. listening to music) and were not significantly correlated with memory retention after 1 day (Bonferroni corrected; see Supplements, Additional file 1: Table 1). One person reported having temporarily fallen asleep during the wakeful resting condition. Excluding the participant from analyses did not change our patterns of results in the ‘Memory performance’ section. On a Likert scale between 1 and 6, participants indicated that, on average, they liked the musical excerpts (*M* = 4.39, SD = 1.27). Nineteen participants indicated that they knew at least one excerpt and 2 participants were not sure. Thirteen participants related at least one music excerpt with an emotional event. All of them indicated that this event had a positive connotation.

The results of Experiment 1 indicate that a brief period of wakeful resting immediately after encoding a word list seems to have a similar impact on memory retention over 1 day as a brief period of listening to music. This finding partly resonates with those of Judde and Rickard (2010) showing that emotionally arousing music affected memory when presented 20 min after encoding a word list but not immediately or 45 min after encoding a word list, in addition to the findings of Rickard et al. (2012), which indicate that they did not find a significant post-encoding condition*memory performance interaction effect (but see also the studies of Martini et al., 2020; Varma et al., 2017).

In a second experiment, we exploratively tested a potential modulating effect of the presented word lists. In Experiment 1, we used relatively concrete words like “cup”, “crane”, or “umbrella”. Findings indicate that memory performance generally increases from abstract words (e.g. conscience, truth) to concrete words. One hypothesis for this effect is that concrete words are represented in a double coding, a verbal code and an images code which can have additive effects resulting in better memory for concrete words than for less concrete words (for a comprehensive overview, see Paivio, 1986; but see also, e.g. Fliessbach et al., 2006; Welcome et al., 2011). We assumed that the encoding of concrete words potentially resulted in stronger memory traces less prone to post-encoding consolidation interference which resulted in similar memory retention scores in the wakeful resting condition and listening to music condition in Experiment 1. In Experiment 2, therefore, we contrasted the concrete word lists of Experiment 1 by constructing word lists consisting of words low in imageability ratings. If the post-encoding activity of wakeful resting and listening to music have similar effects on memory retention, like in Experiment 1, this effect should be found independently of the concreteness of the to-be-encoded words. However, if less concrete words are represented in weaker memory traces more prone to consolidation interference, as expected (Creery et al., 2015; Diekelmann et al., 2010; Denis et al., 2021; Schapiro et al., 2018; Tambini et al., 2018), we should find differences in 1-day memory retention performance in dependence of whether encoding was followed by wakefully resting or listening to music.

## Experiment 2

In Experiment 2, we compared how a 6 min wakeful-resting period after encoding and a 6 min listening to music period after encoding affects memory retention over 1 day for less concrete words.

### Method

#### Participants

Forty-nine university students (35 female, mean age = 22.83 years, SD = 3.41 years, age range = 18–37 years) took part in the experiment in exchange for course credit. All participants gave informed consent to take part in the study.

#### Materials and procedure

The basic experimental procedure was the same as in Experiment 1 (see Method section). The two word lists consisted again of 15 German words each. However, this time words were taken from the Berlin Affective Word List Reloaded (BAWL-R; Võ et al., 2009). The BAWL–R is a list of over 2,900 German words which were rated regarding valence [7-point scale ranging from -3 (very negative) through 0 (neutral) to + 3 (very positive)], arousal [5-point scale ranging from 1 (low arousal) to 5 (high arousal)], and imageability [7-point imageability scale ranged from 1 (low imageability) to 7 (high imageability)]. We selected only words from the BAWL-R database falling within ± 1SD of the reported mean BAWL-R rating scores for the dimensions valence (*M* = 0.24, SD = 1.02) and arousal (*M* = 2.54, SD = 1.05). For the dimension imageability (*M* = 1.87, SD = 1.05) the reported mean rating scores should be as low as possible. All selected words had a mean imageability BAWL-R rating score < 2.2. Words were monosyllabic, bisyllabic, or trisyllabic and consisted of five to nine letters (e.g. “Ahnung[hunch]”, “Reform[reform]”, “Sitte[custom]”, “Zweck[purpose])”. Word lists were created such that each word started with a different first letter. Words were presented one by one, in a pseudo-randomized order, for 2 s in the middle of the screen (black against a white background). Participants were tested in single testing sessions. Delayed recall performance was collected online (see Judde & Rickard, 2010). Participants’ heart rate was measured during Session 1 (these data are not presented in the present paper). The order of the two post-encoding activity conditions was counterbalanced across participants. Each participant had a partition on her/his right, left, and front. During wakeful resting, participants wore capsule earmuffs.

#### Analysis of the data

Scoring and statistical analyses of participants’ memory performance, subjective rehearsal ratings, emotional arousal ratings, and thought activity were the same as in Experiment 1. Descriptive statistics and Spearman correlations between the ARSQ dimensions and our mind wandering items with memory retention over 1 day are provided in the Supplements (Additional file 1: Table 2).

Statistical analyses were conducted using JASP (JASP Team, 2020). The alpha level was set at < 0.05.

#### Memory performance

From six participants, which took part in Session 1, but not in Session 2, we imputed the missing 1-day recall values using the predictive mean matching method in the mice package (v3.14.0; van Buuren & Groothuis-Oudshoorn, 2011). One participant was excluded from further analyses laying 3 SD above the mean 1-day delayed recall performance. The finally analysed data set consisted of forty-eight participants (34 female, mean age = 22.82 years, SD = 3.45 years, age range = 18–37 years). Twenty-five participants were in the ‘first wakeful resting-then listening to music’ condition and 23 participants in the ‘first listening to music-then wakeful resting’ condition.

### Results

#### Memory performance

**Fig. 2:**
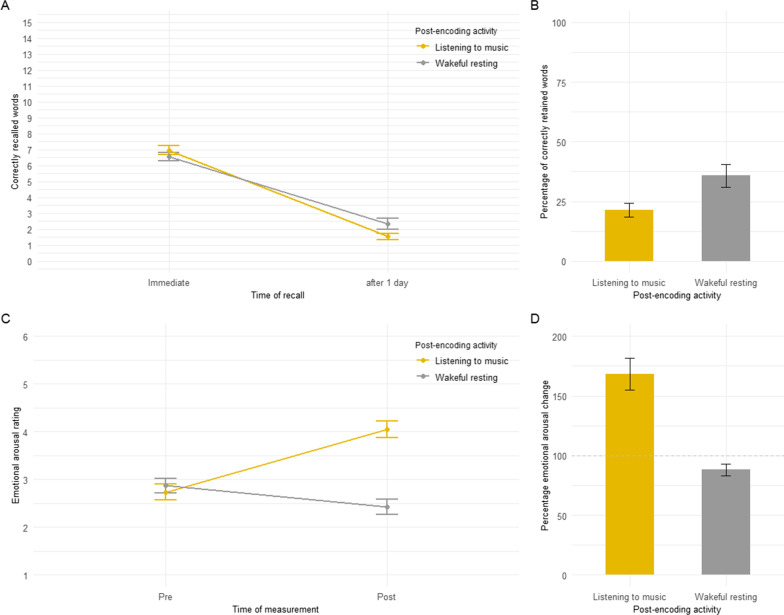
**A** Mean number of words correctly recalled (out of 15 per word list) as a function of the post-encoding activity (wakeful resting [grey] vs. listening to music [yellow]) and time of recall (immediate vs. after 1 day). **B** Mean percentage-retention scores as a function of post-encoding activity (wakeful resting [grey] vs. listening to music [yellow]). Percentage-retention scores were calculated by dividing the number of correctly recalled words in Session 2 by the number of correctly recalled words in Session 1 and multiplying the quotient by 100. **C** Mean emotional arousal ratings as a function of the post-encoding activity (wakeful resting [grey] vs. listening to music [yellow]) and time of measurement (before learning the word list [pre] vs. after the post-encoding activity [post]). **D** Percentage emotional arousal change scores as a function of post-encoding activity (wakeful resting [grey] vs. listening to music [yellow]). Percentage emotional arousal change scores were calculated by dividing participant’s emotional arousal rating before encoding a word list divided by the emotional arousal rating after the respective post-encoding activity (wakeful resting vs. listening to music) multiplied by 100. Dashed helpline: 100% indicates no change in the mean pre- to post emotional arousal ratings. Error bars represent standard errors of the mean

Mean memory performance at the two recall times in the two post-encoding activity conditions (wakeful resting vs. listening to music) is presented in Fig. 2A, which depicts the raw number of words recalled, and in Fig. 2B, which depicts the percentage-retention scores. As shown in Fig. 2B, participants’ retention scores were significantly higher after 1 day if word list encoding was followed by a brief period of wakeful resting than if it was followed by listening to music, *t*(47) = 2.84, *p* = 0.007, *d* = 0.410. The order of the post-learning condition (first wakeful resting-then listening to music vs. first listening to music-then wakeful resting) revealed no significant effect (*p* > 0.20). The same pattern of results was obtained when the raw number of words recalled was analysed (see Fig. 2A and Supplements, Additional file 2: Experiment 2 - Analyses of the raw number of words recalled).

#### Subjective rehearsal ratings

Participants’ rehearsal ratings indicate that words were only rarely rehearsed during the respective post-encoding activity condition (wakeful resting: *M* = 2.04, SD = 1.7; listening to music: *M* = 1.47, SD = 0.89). The difference in rehearsal ratings between the two post-encoding activity conditions was statistically significant, *W* = 234.00, *p* = 0.003, *r*_rank-biserial_ = 0.696. Rehearsal ratings in the wakeful resting condition were not significantly correlated with memory retention in the wakeful resting condition, *r*_Spearman_ = 0.276, *p* = 0.067 (see Additional file 3: Fig. 3, Supplements). Rehearsal ratings in the listening to music condition were not significantly correlated with memory retention in the listening to music condition, *r*_Spearman_ = 0.173, *p* = 0.256 (see Additional file 3: Fig. 3, Supplements).

#### Subjective arousal ratings

Mean arousal ratings at the two recall times in the two post-encoding activity conditions (wakeful resting vs. listening to music) are presented in Fig. 2C, which depicts the raw emotional arousal ratings, and in Fig. 2D, which depicts the emotional arousal change scores (for a description see Methods section). As shown in Fig. 2D, emotional arousal change increased in the listening to music condition (> 100%) and decreased in the wakeful resting condition (< 100%), *W* = 20.00, *p* < 0.001, *r*_rank-biserial_ = − 0.951. Emotional arousal change in the wakeful resting condition was not significantly correlated with memory retention in the wakeful resting condition, *r*_Spearman_ = − 0.02, *p* = 0.884, and emotional arousal change in the listening to music condition was not significantly correlated with memory retention in the listening to music condition, *r*_Spearman_ = 0.05, *p* = 0.722.

Participants’ thought activity was diverse within and between the respective post-encoding activity condition (wakeful resting vs. listening to music) and were not significantly correlated with memory retention after 1 day (Bonferroni corrected; see Supplements, Additional file 1: Table 2). No person reported having fallen temporarily asleep during the wakeful resting condition. On a Likert scale between 1 and 6, participants indicated that they liked the musical excerpts on average 3.98 (SD = 1.27). Twenty-seven participants indicated that they knew at least one excerpt. Twenty-two participants related at least one music excerpt with an emotional event. All of them indicated that this event had positive connotations.

## Experiment 1 and Experiment 2: combined memory performance results

Acknowledging that Experiment 1 and Experiment 2 used different populations and were conducted at different times with variations in the methodological approach, we conducted a cross-experimental statistical analysis of participants’ memory performance (immediate recall performance and memory retention over 1 day).

For the analysis of participants’ immediate recall performance, we conducted a mixed analysis of variance with post-encoding activity condition (wakeful resting vs. listening to music) as within-subject factor and experiment (Experiment 1 vs. Experiment 2) as between-subjects factor. Statistical analyses revealed no significant effect of post-encoding activity condition, *F*(1,86) = 0.75, *p* = 0.391, *η*_*p*_^*2*^ = 0.009, overall participants immediately recalled a similar amount of words in the wakeful resting condition and listening to music condition. A significant effect of experiment, *F*(1,86) = 35.82, *p* < 0.001, *η*_*p*_^*2*^ = 0.294, indicated that overall participants immediately recalled significantly more words in Experiment 1 than in Experiment 2. There was no significant interaction between immediate recall performance and experiment, *F*(1,86) = 0.96, *p* = 0.330, *η*_*p*_^*2*^ = 0.011.

For the analysis of participants’ retention performance over 1 day, we conducted a mixed analysis of variance with post-encoding activity condition (wakeful resting vs. listening to music) as within-subject factor and experiment (Experiment 1 vs. Experiment 2) as between-subjects factor. Statistical analyses revealed a significant effect of post-encoding activity condition, *F*(1,86) = 6.71, *p* = 0.011, *η*_*p*_^*2*^ = 0.072, overall participants retained more words in the wakeful resting condition than listening to music condition. A significant effect of experiment, *F*(1,86) = 21.62, *p* < 0.001, *η*_*p*_^*2*^ = 0.201, revealed that overall participants retained more words in Experiment 1 than in Experiment 2. There was no significant interaction between post-encoding activity condition and experiment, *F*(1,86) = 2.74, *p* = 0.101, *η*_*p*_^*2*^ = 0.031.

## Discussion

The present study investigated the effects of wakeful resting and listening to music immediately after encoding a word list on memory. In Experiment 1, we demonstrate that participants retained a similar amount of words after 1 day in the wakeful resting condition and listening to music condition. In Experiment 2, where participants were required to encode words low in imageability, participants retained more words after 1 day in the wakeful resting condition compared to the listening to music condition.

The question arises, why should less concrete words be more affected and more concrete words be less affected by wakeful resting and listening to music? Studies indicate that memory performance improves from abstract words to concrete words (Paivio, 1986). It is easier to maintain words like “duck” or “table” compared to “truth” or “hunch”. Theoretical explanations for this ‘concreteness effect’ assume that concrete words form a dual code, i.e. a verbal and sensory code, and/or have a more accessible semantic network than less concrete words (Fliessbach et al., 2006). A meta-analytical review of neuroimaging studies by Wang et al. (2010) brought further insight showing that less concrete words engage more the verbal system while concrete words engage more the perceptual system—via mental imagery. It seems reasonable to assume that more concrete words are associated with more stable memory traces than less concrete words. This might explain why we found that more concrete words (Experiment 1) were similarly affected by the two post-encoding activities, whereas only less concrete words (Experiment 2) could illuminate differences in how the two post-encoding activities affect memory. Our results from Experiment 2 support the view that less concrete words benefit from a state during which attention to external sensory input is reduced (Craig et al., 2014; Dewar et al., 2012; Mednick et al., 2011; Robertson, 2012; Wamsley, 2019; Wixted, 2005). Furthermore, consolidation has been associated with the reactivation of recent experiences (Dudai et al., 2015; Tambini & Davachi, 2019; Tambini et al., 2010). Wakeful resting might just provide conditions of minimal interference, during which previously encoded words can be reactivated more often than is possible during periods filled with listening to music. An increase of these automatic reactivations during wakeful resting could allow the memory traces of less concrete words to be strengthened to a higher degree than is possible during listening to music (see also Schapiro et al., 2018). Potentially, more concrete words are represented in more stable memory traces than less concrete words because of dual coding and/or a better embedding into a pre-existing semantic neural network. It could be speculated therefore that a disruption of automatic reactivations, in our experiments by listening to music, might have interfered less with memory consolidation processes for more concrete words as these memory traces needed fewer reactivations to build more stable memory representations. This assumption resonates with findings from studies showing that our brain can prioritize memories to be consolidated. There is evidence that memories are more likely to be reactivated during a post-encoding awake rest period when associated with reward (Gruber et al., 2016) or fear (de Voogd et al., 2016). There is further evidence that memories relevant for future behaviour (Wilhelm et al., 2011), emotionally salient stimuli (Hu et al., 2006), and weaker memories benefit more from a period of sleep (Denis et al., 2021; Diekelmann et al., 2010; Drosopoulos et al., 2007; Petzka et al., 2021; Schapiro et al., 2018). Furthermore, weaker memories seem to benefit more from a targeted reactivation than stronger memories (Cairney et al., 2014; Creery et al., 2015; Tambini et al., 2017). At least for sleep-related memory improvements, it is assumed that slow oscillatory activity supports the communication between learning relevant brain areas in the neocortex and the hippocampus and the consolidation of these new memories (Sirota & Buzsáki, 2005). This latter view is in-line with findings from Brokaw et al. (2016) showing that improved memory was associated with an increase in slow oscillatory brain activity during a wakeful resting state immediately after encoding new information. Altogether, we suggest that less concrete words, low in imageability, are associated with weaker memory traces which can benefit more from a brief period of wakeful resting and are more prone to interference inducted by listening to music, compared to more concrete words, high in imageability.

It could be argued that the beneficial effect of wakeful resting in Experiment 2 was due to an increased opportunity to rehearse the just-encoded information. We found that participants rehearsed words significantly more often during wakeful resting than listening to music, however, mean rehearsal rates were very low and not significantly related to memory retention in the respective post-encoding activity condition. Our findings resonate with others showing that difficult to rehearse verbal material and thinking about the just-encoded verbal information during wakeful resting is not or only weakly related to memory retention (e.g. Brokaw et al., 2016; Dewar et al., 2014). Nevertheless, we cannot exclude the possibility that some rehearsal activity might have had a modulating effect on memory also in our experiments. Inspection of Additional file 3: Fig. 3 (Supplements) shows a trend in both experiments to the effect that rehearsal is positively related with the number of words retained after 1 day. Conceptually, it makes sense to assume that rehearsal is an effective cognitive strategy to prevent forgetting and stabilize weakly represented information. Especially a brief period of wakeful resting can therefore represent an ideal opportunity to rehearse the just encoded information. However, results stemming from a single post-conditional question, as it was the case in our study, have to be considered cautiously for several reasons. First, a subjective post-conditional rating can only be a rough estimate of rehearsal activity taking place over a 6 min interval—for example, fluctuations of rehearsal activity over time cannot be represented by it. Second, the rehearsal activity rating was heavily subjective—for example, a rating of “3” on the rehearsal activity scale will hardly bear the same meaning for all participants. Third, participants were not provided with clear definitions of rehearsal activity which potentially confounded the impact of different rehearsal types like explicit/intentional rehearsal versus spontaneous/unintentional rehearsal.

One remaining open question is what exactly could lead to potential consolidation interfering effects in the music condition. In our study, we included musical excerpts from the genres pop, rock, and dance, with vocals, high in musical tempo. It is possible that the interfering effect of listing to music might be due to these two latter, mutually not exclusive factors. Regarding vocals, evidence exists that memory interference can occur between information that is similar in its content, for instance when verbal encoding material is processed simultaneously or in short succession with verbal distractor material, resulting in a decreased memory performance (e.g. Dewar et al., 2007; Jones & Macken, 1993; Müller & Pilzecker, 1900). Thus, it is conceivable that vocals included in our musical excerpts eventually interfered with consolidation leading to a decreased memory performance after 1 day. In our study, participants encoded words in their mother tongue (German) and listened to musical pieces including vocals in participants’ second language (English). Words from the word lists were thereby not contained in the text of the vocals of the musical pieces. Therefore, we would assume that the encoded words and words from the vocal text were not likely to affect each other. However, a potential translation process itself, i.e. when participants tried to translate the vocals into their mother tongue, which can be described as cognitive demanding activity, could have led to consolidation interfering effects (but see Martini et al., 2020) in addition to a potential interfering effect due to a semantic overlap of encoded words and vocals.

Regarding the musical tempo, evidence exists that musical tempo manipulations induce changes in the arousal state (Balch & Lewis, 1996), and are associated with expressions of activity, excitement, and surprise (e.g. Gabrielsson & Lindström, 2001; Scherer & Oshinsky, 1977; van der Zwaag et al., 2011). In our study musical tempo was over 150 bpm per excerpt, thus it is reasonable to assume that this might have increased arousal, which in turn could have had a detrimental effect on memory consolidation (see also McGaugh, 2015). However, there is contrasting evidence on the effect of tempo in increasing arousal. In fact, in the study by Judde and Rickard (2010), findings suggested that the musical pieces‘ valence (i.e. negative, positive) was able to modulate arousal independently from the musical tempo. Specifically, negative excerpts were slower than 150 bpm but were anyway able to increase arousal, suggesting that tempo alone might be not sufficient in influencing memory consolidation. Whether in the current study the tempo alone was indirectly linked to a detrimental effect in memory consolidation (through increased arousal), remains thus to be determined.

Potential interfering effects in the listening to music condition might also have come from an increased emotional arousal state or a changed mind wandering activity during the period participants listened to music. Studies indicate that music is a potential means to modulate emotional arousal and that emotional arousal, in turn, can modulate memory consolidation processes (McGaugh, 2015). The findings of the few studies so far investigating the impact of emotional arousing music following learning found tendentially positive effects on memory (Greene et al., 2010; Judde & Rickard, 2010; but see Experiment 2 of Rickard et al., 2012 showing that relaxing music also can have consolidation interfering effects on emotional story elements). The findings of these studies would therefore suggest that listening to emotionally arousing music following learning should rather have effects supporting consolidation than effects interfering with consolidation, which is contrary to our findings in Experiment 2. However, it is also suggested that the effects of emotional arousal on memory consolidation depend on the emotional arousal level. More concretely, it is assumed that the relationship between emotional arousal and memory follows an inverted U-shaped function (see McGaugh, 2015, 2018, but also Mather & Sutherland, 2011). Accordingly, high emotional arousal states can under certain conditions also lead to poorer memory. Our findings indicate that there was a significant change in subjective emotional arousal ratings from before learning a word list to the rating after the respective post-encoding activity, with a significantly higher proportional change in the listening to music condition (see Figs. 1D and 2D). However, we found that these proportional changes in emotional arousal were not related to the number of words retained after 1 day in the listening to music condition, which potentially would indicate that emotional arousal was not the main driving factor for our results. This issue should have been further tested by more concrete phrased items and in the view of the fact that participant ratings are subjective, a guesstimate of a dynamic process which can vary over time, and which are contingently biased by the condition itself.

Regarding participants’ mind wandering activity, as outlined above, studies indicate that autobiographical retrieval induced by concrete external cues can impair memory consolidation (Craig et al., Varma et al., 2018). It stands to reason that the musical pieces induced the retrieval of concrete autobiographical events or imaginations which might have interfered with the consolidation of the word lists. However, even though our results indicate that the musical pieces induced the retrieval of positive concrete events, at least in some participants, and thought activity during listening to music was highly diverse (see Additional file 1: Tables 1 and 2, Supplements), we did not find significant correlations (after Bonferroni correction) with the number of words retained after 1 day (see also Brokaw et al., 2016). Potentially, more focussed research questions delineating specific mind wandering activities together with a higher experimental power might reveal interesting new findings in future studies.

Those few existing studies investigating the impact of listening to music immediately following learning found no or positive effects of listening to music on memory (Greene et al., 2010; Judde & Rickard, 2010; Rickard et al., 2012). It is important to note that our experimental approach differed from those studies by at least two major aspects which make different outcomes plausible. First and most relevant, our main goal was to delineate listening to music from a different post-encoding activity, here wakeful resting. This experimental approach differs from the study of Judde and Rickard (2010) who did not compare a listening to music condition to another condition. Their main research focus was the timing of the music intervention after encoding a word list, and their control group was not exposed to any post-encoding activity intervention. Similarly, Greene et al. (2010) varied post-encoding emotional states by means of exposure to music that varied in both mood and arousal dimensions and measured their impact on visual recognition memory. Both studies, therefore, did not compare a listening to music condition to another post-encoding activity. From these studies, it is therefore unclear whether the results are due to music per se. The study of Rickard et al., (2012, Experiment 2) investigated the impact of listening to music to a no-music condition of equal length. However, the no-music condition differed fundamentally from our wakeful resting condition. In the no-music condition of Rickard et al.’s (2012) experiment, participants heard background sounds in a busy café, which consisted of footsteps and people conversing at a distance. In our study, we compared an eyes-closed wakeful resting condition with an eyes-closed listening to music condition of equal length. In our wakeful resting condition, auditory sensory input was at a minimum while auditory sensory input in the control condition of Rickard et al. (2012) seemed to be constantly high. Background sound in Rickard et al.’s (2012) control condition potentially interfered with memory consolidation which could have masked the true effects of the listening to music conditions. This view is supported by findings showing that concrete sound cues, which elicit autobiographical thinking have been shown to impair the influence of rest on memory (Craig et al., 2014; Varma et al., 2018).

Our study has several additional limitations. First, our findings are limited to unrelated words as encoding material. Second, it might be interesting to test the impact of resting and music in dependence on a reference condition, e.g. performing a cognitively demanding task (e.g. Dewar et al., 2007). This would be especially interesting in light of the finding of Experiment 1 as only with a third post-encoding condition it can be tested whether the two post-encoding activities were similar in their effect on memory because they both had a consolidation supporting effect, or whether they both rather interfered with consolidation (see also Kuschpel et al., 2015).

To conclude, this is the first study comparing the effects of wakeful resting and listening to music following encoding on memory. Our findings indicate that the effect of the post-encoding activity depends on the concreteness of the verbal encoding material—if the verbal material is concrete resting and music have similar effects on memory, if the verbal material is less concrete memory benefits from a brief period of rest and is comparably impaired by a brief period of listening to music.

## Supplementary Information

**Additional file 1**. Descriptive statistics of participants’ thought activity during wakeful resting/listening to music and their correlations with memory retention performance after 1 day for Experiment 1 and Experiment 2.

**Additional file 2**. Analyses of the raw number of words recalled for Experiment 1 and Experiment 2.

**Additional file 3**.** Fig. 3**. Scatterplots for the correlation between memory retention and participants‘ rehearsal ratings plotted separately for the post-encoding activity condition (wakeful resting [grey regression lines] vs. listening to music [yellow regression lines]) and experiment (Experiment 1 vs. Experiment 2).

## Data Availability

The datasets used and/or analysed during the current study are available from the corresponding author on reasonable request.
