# Supplementary material for: Wakeful resting and listening to music contrast their effects on verbal long-term memory in dependence on word concreteness
Source: Cogn Res Princ Implic. 2022 Sep 3;7:80. doi: 10.1186/s41235-022-00415-4 (PMC9440969; doi:10.1186/s41235-022-00415-4)
Supplement: Supplementary file 1 — Additional file 1. Descriptive statistics of participants’ thought activity during wakeful resting/listening to music and their correlations with memory retention performance after 1 day for Experiment 1 and Experiment 2. [file 41235_2022_415_MOESM1_ESM.docx]

**Supplements**

**Experiment 1 – Thought activity**

Thought activities were not significantly correlated with 1-day memory retention performance in the respective post-encoding activity condition (wakeful resting vs. listening to music). *p*-values were Bonferroni corrected (*p* <.003).

Table 1

*Descriptive statistics of participants’ thought activity during wakeful resting/listening to music and their correlations with memory retention performance after 1 day.*

|  |  |  | | ***Spearman's rho*** | | ***p*** |  | ***M*** |  | ***SD*** |
| --- | --- | --- | --- | --- | --- | --- | --- | --- | --- | --- |
| **Wakeful resting condition** |  |  |  |  |  |  |  |  |  |  |
| Discontinuity of Mind^^^ | - | Memory retention |  | -0.175 |  | 0.279 |  | 2.46 |  | 0.74 |
| Theoy of mind^^^ | - | Memory retention |  | 0.103 |  | 0.527 |  | 2.86 |  | 1.15 |
| Self^ | - | Memory retention |  | -0.014 |  | 0.933 |  | 3.39 |  | 0.87 |
| Planning^^^ | - | Memory retention |  | -0.257 |  | 0.109 |  | 3.35 |  | 1.15 |
| Sleepiness^^^ | - | Memory retention |  | -0.024 |  | 0.882 |  | 2.73 |  | 1.11 |
| Comfort^^^ | - | Memory retention |  | 0.173 |  | 0.286 |  | 3.38 |  | 0.78 |
| Somatic Awareness^^^ | - | Memory retention |  | 0.028 |  | 0.864 |  | 2.59 |  | 1.16 |
| Health Concern^^^ | - | Memory retention |  | -0.093 |  | 0.568 |  | 1.50 |  | 0.64 |
| Visual thought^^^ | - | Memory retention |  | -0.006 |  | 0.969 |  | 3.46 |  | 1.27 |
| Verbal thought^^^ | - | Memory retention |  | -0.156 |  | 0.335 |  | 2.50 |  | 0.83 |
| Past | - | Memory retention |  | 0.073 |  | 0.653 |  | 2.73 |  | 1.41 |
| Present | - | Memory retention |  | 0.045 |  | 0.783 |  | 3.95 |  | 0.96 |
| Future | - | Memory retention |  | -0.203 |  | 0.210 |  | 3.83 |  | 1.34 |
| Sorrow | - | Memory retention |  | -0.158 |  | 0.329 |  | 3.15 |  | 1.41 |
| Happiness | - | Memory retention |  | -0.255 |  | 0.112 |  | 2.93 |  | 1.29 |
| Anger | - | Memory retention |  | -0.103 |  | 0.528 |  | 1.98 |  | 1.12 |
| Sadness | - | Memory retention |  | -0.052 |  | 0.751 |  | 2.00 |  | 1.26 |
| **Listening to music condition** |  |  |  |  |  |  |  |  |  |  |
| Discontinuity of Mind^^^ | - | Memory retention |  | 0.026 |  | 0.875 |  | 2.21 |  | 0.89 |
| Theoy of mind^^^ | - | Memory retention |  | -0.086 |  | 0.599 |  | 2.56 |  | 1.17 |
| Self^ | - | Memory retention |  | 0.063 |  | 0.700 |  | 2.97 |  | 0.94 |
| Planning^^^ | - | Memory retention |  | -0.066 |  | 0.686 |  | 2.39 |  | 1.22 |
| Sleepiness^^^ | - | Memory retention |  | -0.000 |  | 0.997 |  | 1.59 |  | 0.74 |
| Comfort^^^ | - | Memory retention |  | -0.107 |  | 0.512 |  | 3.48 |  | 0.73 |
| Somatic Awareness^^^ | - | Memory retention |  | 0.097 |  | 0.553 |  | 2.08 |  | 0.84 |
| Health Concern^^^ | - | Memory retention |  | -0.005 |  | 0.974 |  | 1.24 |  | 0.52 |
| Visual thought^^^ | - | Memory retention |  | 0.026 |  | 0.875 |  | 3.47 |  | 1.20 |
| Verbal thought^^^ | - | Memory retention |  | 0.122 |  | 0.452 |  | 2.00 |  | 0.82 |
| Past | - | Memory retention |  | 0.055 |  | 0.737 |  | 2.88 |  | 1.44 |
| Present | - | Memory retention |  | 0.245 |  | 0.128 |  | 3.40 |  | 1.26 |
| Future | - | Memory retention |  | -0.167 |  | 0.302 |  | 3.48 |  | 1.24 |
| Sorrow | - | Memory retention |  | 0.023 |  | 0.890 |  | 2.53 |  | 1.43 |
| Happiness | - | Memory retention |  | -0.202 |  | 0.211 |  | 3.23 |  | 1.29 |
| Anger | - | Memory retention |  | -0.002 |  | 0.988 |  | 1.80 |  | 1.04 |
| Sadness | - | Memory retention |  | 0.048 |  | 0.768 |  | 1.80 |  | 1.02 |

* <.003 (Bonferroni corrected: .05/17). ^^^ = Amsterdam Resting State Questionnaire (ARSQ, Diaz et al., 2014).

**Experiment 2 – Thought activity**

Thought activities were not significantly correlated with 1-day memory retention performance in the respective post-encoding activity condition (wakeful resting vs. listening to music). *p*-values were Bonferroni corrected (*p* <.003).

Table 2

*Descriptive statistics of participants’ thought activity during wakeful resting/listening to music and their correlations with memory retention performance after 1 day.*

|  |  |  | | ***Spearman's rho*** | | | ***p*** | |  | | ***M*** | |  | | ***SD*** | |  |
| --- | --- | --- | --- | --- | --- | --- | --- | --- | --- | --- | --- | --- | --- | --- | --- | --- | --- |
| **Wakeful resting condition** |  |  |  | |  |  | |  | |  | |  | |  | |  | |
| Discontinuity of Mind^^^ | - | Memory retention |  | | 0.078 |  | | 0.610 | |  | | 2.30 | |  | | 0.83 | |
| Theoy of mind^^^ | - | Memory retention |  | | -0.424 |  | | 0.004 | |  | | 2.99 | |  | | 1.07 | |
| Self^ | - | Memory retention |  | | -0.020 |  | | 0.896 | |  | | 3.10 | |  | | 1.13 | |
| Planning^^^ | - | Memory retention |  | | -0.219 |  | | 0.148 | |  | | 2.59 | |  | | 1.04 | |
| Sleepiness^^^ | - | Memory retention |  | | -0.142 |  | | 0.354 | |  | | 2.05 | |  | | 1.06 | |
| Comfort^^^ | - | Memory retention |  | | -0.012 |  | | 0.938 | |  | | 3.30 | |  | | 0.65 | |
| Somatic Awareness^^^ | - | Memory retention |  | | 0.147 |  | | 0.335 | |  | | 3.03 | |  | | 1.14 | |
| Health Concern^^^ | - | Memory retention |  | | 0.079 |  | | 0.605 | |  | | 1.36 | |  | | 0.55 | |
| Visual thought^^^ | - | Memory retention |  | | -0.029 |  | | 0.848 | |  | | 3.17 | |  | | 1.37 | |
| Verbal thought^^^ | - | Memory retention |  | | -0.310 |  | | 0.039 | |  | | 2.04 | |  | | 0.77 | |
| Past | - | Memory retention |  | | -0.120 |  | | 0.431 | |  | | 2.87 | |  | | 1.36 | |
| Present | - | Memory retention |  | | 0.132 |  | | 0.386 | |  | | 3.47 | |  | | 1.34 | |
| Future | - | Memory retention |  | | -0.198 |  | | 0.193 | |  | | 3.56 | |  | | 1.37 | |
| Sorrow | - | Memory retention |  | | -0.139 |  | | 0.364 | |  | | 2.91 | |  | | 1.35 | |
| Happiness | - | Memory retention |  | | -0.336 |  | | 0.024 | |  | | 2.47 | |  | | 1.14 | |
| Anger | - | Memory retention |  | | -0.073 |  | | 0.631 | |  | | 1.96 | |  | | 1.22 | |
| Sadness | - | Memory retention |  | | -0.093 |  | | 0.543 | |  | | 1.89 | |  | | 1.15 | |
| **Listening to music condition** |  |  |  | |  |  | |  | |  | |  | |  | |  | |
| Discontinuity of Mind^^^ | - | Memory retention |  | | 0.037 |  | | 0.807 | |  | | 1.85 | |  | | 0.73 | |
| Theoy of mind^^^ | - | Memory retention |  | | -0.184 |  | | 0.226 | |  | | 2.49 | |  | | 1.15 | |
| Self^ | - | Memory retention |  | | 0.063 |  | | 0.689 | |  | | 2.67 | |  | | 0.95 | |
| Planning^^^ | - | Memory retention |  | | -0.114 |  | | 0.466 | |  | | 1.94 | |  | | 1.04 | |
| Sleepiness^^^ | - | Memory retention |  | | 0.073 |  | | 0.643 | |  | | 1.34 | |  | | 0.54 | |
| Comfort^^^ | - | Memory retention |  | | 0.121 |  | | 0.439 | |  | | 3.43 | |  | | 0.96 | |
| Somatic Awareness^^^ | - | Memory retention |  | | 0.041 |  | | 0.792 | |  | | 2.23 | |  | | 1.02 | |
| Health Concern^^^ | - | Memory retention |  | | -0.008 |  | | 0.961 | |  | | 1.21 | |  | | 0.35 | |
| Visual thought^^^ | - | Memory retention |  | | 0.186 |  | | 0.233 | |  | | 1.21 | |  | | 1.24 | |
| Verbal thought^^^ | - | Memory retention |  | | -0.082 |  | | 0.600 | |  | | 3.34 | |  | | 0.71 | |
| Past | - | Memory retention |  | | 0.310 |  | | 0.038 | |  | | 2.71 | |  | | 1.38 | |
| Present | - | Memory retention |  | | -0.071 |  | | 0.641 | |  | | 2.73 | |  | | 1.39 | |
| Future | - | Memory retention |  | | -0.102 |  | | 0.504 | |  | | 2.80 | |  | | 1.42 | |
| Sorrow | - | Memory retention |  | | 0.002 |  | | 0.992 | |  | | 1.84 | |  | | 1.07 | |
| Happiness | - | Memory retention |  | | -0.023 |  | | 0.879 | |  | | 2.87 | |  | | 1.33 | |
| Anger | - | Memory retention |  | | 0.258 |  | | 0.087 | |  | | 1.69 | |  | | 1.10 | |
| Sadness | - | Memory retention |  | | -0.128 |  | | 0.401 | |  | | 1.49 | |  | | 1.08 | |

* <.003 (Bonferroni corrected: .05/17). ^^^ = Amsterdam Resting State Questionnaire (ARSQ, Diaz et al., 2014).
