# Supplementary material for: Wakeful resting and listening to music contrast their effects on verbal long-term memory in dependence on word concreteness
Source: Cogn Res Princ Implic. 2022 Sep 3;7:80. doi: 10.1186/s41235-022-00415-4 (PMC9440969; doi:10.1186/s41235-022-00415-4)
Supplement: Supplementary file 2 — Additional file 2. Analyses of the raw number of words recalled for Experiment 1 and Experiment 2. [file 41235_2022_415_MOESM2_ESM.docx]

**Supplements**

**Experiment 1 - Analyses of the raw number of words recalled (see Figure 1 A)**

Repeated measures ANOVA:

- Recall time (immediate vs. after 1 day): *F*(1,39) = 132.80, *p* < .001, *η_p_^2^* = .773.
- Post-encoding activity (wakeful resting vs. listening to music): *F*(1,39) = .22, *p* = .642, *η_p_^2^* = .006.
- Recall time*post-encoding activity: *F*(1,39) = .648, *p* = .426, *η_p_^2^* = .016.

**Experiment 2 - Analyses of the raw number of words recalled (see Figure 2 A)**

Repeated measures ANOVA:

- Recall time (immediate vs. after 1 day): *F*(1,47) = 513.24, *p* < .001, *η_p_^2^* = .916.
- Post-encoding activity (wakeful resting vs. listening to music): *F*(1,47) = 1.05, *p* = .310, *η_p_^2^* = .022.
- Recall time*post-encoding activity: *F*(1,47) = 7.11, *p* = .010, *η_p_^2^* = .131.
