# Supplementary material for: Wakeful resting and listening to music contrast their effects on verbal long-term memory in dependence on word concreteness
Source: Cogn Res Princ Implic. 2022 Sep 3;7:80. doi: 10.1186/s41235-022-00415-4 (PMC9440969; doi:10.1186/s41235-022-00415-4)
Supplement: Supplementary file 3 — Additional file 3. Fig. 3. Scatterplots for the correlation between memory retention and participants‘ rehearsal ratings plotted separately for the post-encoding activity condition (wakeful resting [grey regression lines] vs. listening to music [yellow regression lines]) and experiment (Experiment 1 vs. Experiment 2). [file 41235_2022_415_MOESM3_ESM.docx]

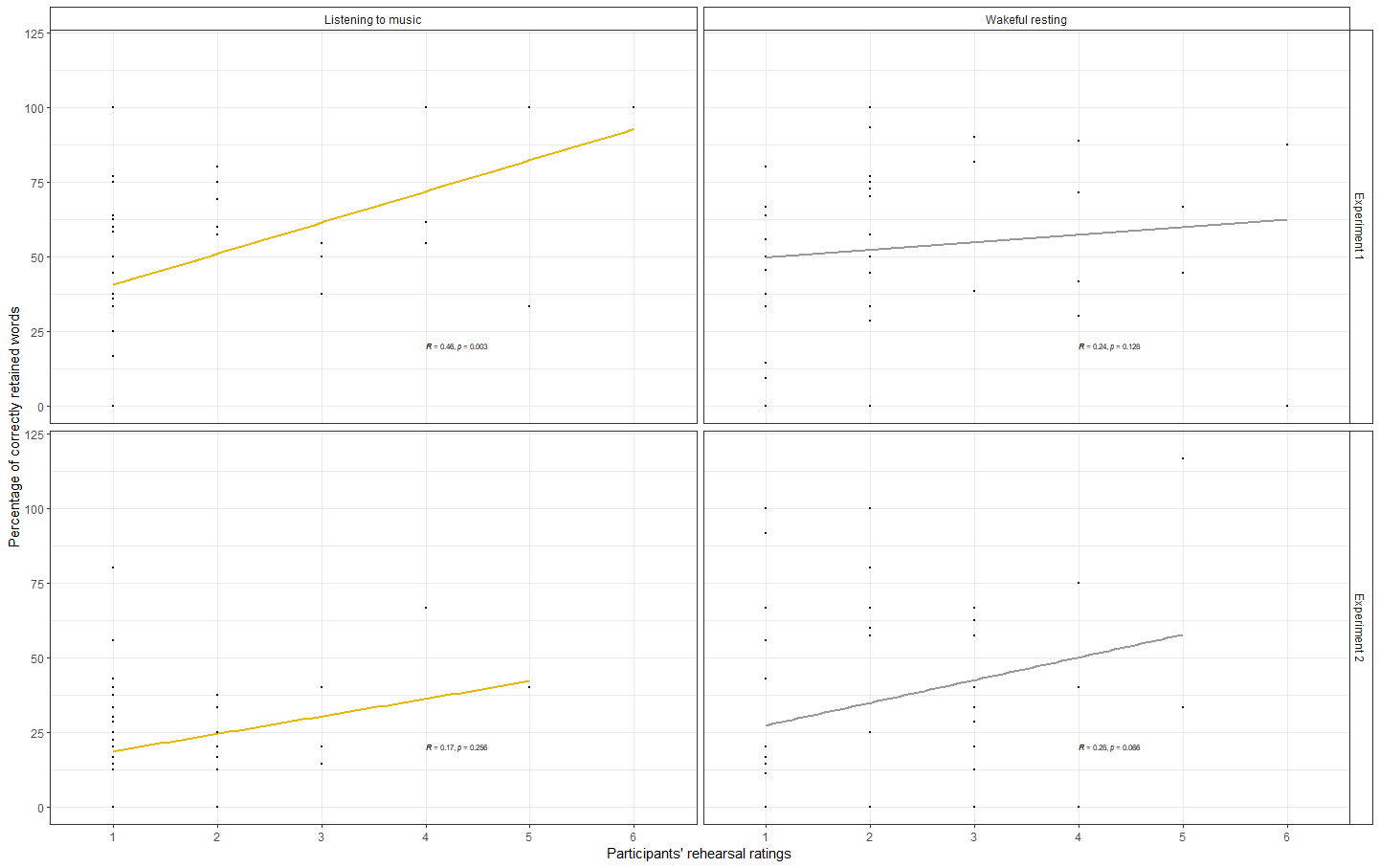


Figure 3. Scatterplots for the correlation between memory retention and participants‘ rehearsal ratings plotted separately for the post-encoding activity condition (wakeful resting [grey regression lines] vs. listening to music [yellow regression lines]) and experiment (Experiment 1 vs. Experiment 2).
